# Phytosterols, Phytostanols, and Lipoprotein Metabolism

**DOI:** 10.3390/nu7095374

**Published:** 2015-09-17

**Authors:** Helena Gylling, Piia Simonen

**Affiliations:** 1 University of Helsinki and Helsinki University Central Hospital, Internal Medicine, Biomedicum Helsinki C 4 22, P.O. BOX 700, 00029 HUS, Helsinki, Finland; 2 University of Helsinki and Helsinki University Central Hospital, Heart and Lung Center, P.O. BOX 700, 00029 HUS, Helsinki, Finland; E-Mail: piia.simonen@hus.fi

**Keywords:** cholesterol absorption, LDL, phytosterol, phytostanol

## Abstract

The efficacy of phytosterols and phytostanols added to foods and food supplements to obtain significant non-pharmacologic serum and low density lipoprotein (LDL) cholesterol reduction is well documented. Irrespective of age, gender, ethnic background, body weight, background diet, or the cause of hypercholesterolemia and, even added to statin treatment, phytosterols and phytostanols at 2 g/day significantly lower LDL cholesterol concentration by 8%–10%. They do not affect the concentrations of high density lipoprotein cholesterol, lipoprotein (a) or serum proprotein convertase subtilisin/kexin type 9. In some studies, phytosterols and phytostanols have modestly reduced serum triglyceride levels especially in subjects with slightly increased baseline concentrations. Phytosterols and phytostanols lower LDL cholesterol by displacing cholesterol from mixed micelles in the small intestine so that cholesterol absorption is partially inhibited. Cholesterol absorption and synthesis have been carefully evaluated during phytosterol and phytostanol supplementation. However, only a few lipoprotein kinetic studies have been performed, and they revealed that LDL apoprotein B-100 transport rate was reduced. LDL particle size was unchanged, but small dense LDL cholesterol concentration was reduced. In subjects with metabolic syndrome and moderate hypertriglyceridemia, phytostanols reduced not only non- high density lipoprotein (HDL) cholesterol concentration but also serum triglycerides by 27%, and reduced the large and medium size very low density lipoprotein particle concentrations. In the few postprandial studies, the postprandial lipoproteins were reduced, but detailed studies with apoprotein B-48 are lacking. In conclusion, more kinetic studies are required to obtain a more complete understanding of the fasting and postprandial lipoprotein metabolism caused by phytosterols and phytostanols. It seems obvious, however, that the most atherogenic lipoprotein particles will be diminished.

## 1. Introduction

### 1.1. What Are Phytosterols and Phytostanols?

Phytosterols and phytostanols are normal bioactive components in plants and in different foods of plant origin. They have similar functions in plants as that of cholesterol in humans. Phytosterols differ from cholesterol by having a different structure in their side chain, whereas phytostanols are 5α-saturated derivatives of phytosterols. These structural changes, even though minor, make cholesterol, phytosterols, and phytostanols differ from each other functionally and metabolically. Phytosterols and phytostanols are not synthesized in the human body so that they are completely derived from dietary sources. Phytosterols and phytostanols are present in vegetable foods, especially in vegetable oils (corn oil, rapeseed (canola) oil, soybean oil, and sunflower oil), nuts, seeds, and cereals [1]. The main food sources for phytosterols are vegetable oils, vegetable-fat spreads and margarines, nuts, cereals and cereal products (bread), and vegetables [2,3]. The main food sources for phytostanols are cereals, especially wheat and rye [1,2,4]. The most abundant phytosterols and phytostanols in human diet are sitosterol, campesterol, sitostanol, and campestanol. In a typical Western diet, the mean daily intake of phytosterols is about 300 mg and that of phytostanols about 30 mg [2,3,4].

The intestinal absorption efficiency of phytosterols and phytostanols is very low compared with that of cholesterol, which is ~50%. The absorption efficiency of phytosterols is less than 2%, and that of phytostanols less than 0.2% [5]. Serum phytosterol concentration is low, less than 24 µmol/L (<1.0 mg/dL), and serum phytostanol concentration is even lower, less than 0.3 µmol/L (<12 µg/dL). In the circulation, phytosterols and phytostanols are carried in lipoproteins similarly to cholesterol, so that they circulate mainly in low density lipoprotein (LDL) (70%–80%) and in high density lipoprotein (HDL) particles (20%–30%) [6]. In detail, in type 2 diabetics with normal weight, good to moderate glucose balance, no insulin therapy, mild to moderate hypercholesterolemia, and normotriglyceridemia, campesterol and sitosterol concentrations were recovered in different lipoproteins as follows: 7%–9% in very low density lipoproteins (VLDL), 3%–4% in intermediate density lipoproteins (IDL), 59%–61% in LDL, and 27%–30% in HDL, respectively [7]. However, proportionately to the respective lipoprotein cholesterol value, the phytosterols and phytostanols were mainly carried in HDL [7,8] and in type 2 diabetes also in the IDL fraction [7]. Phytosterols and phytostanols are taken up into tissues in similar proportions relative to cholesterol, and the ratios of phytosterols and phytostanols to cholesterol in tissues are practically similar to that in serum [9].

The objective of this review was to discuss what is known about lipoprotein metabolism during phytosterol and phytostanol consumption both under fasting conditions and in postprandium to clarify their effects on the whole-body cholesterol metabolism in human subjects.

### 1.2. Phytosterols, Phytostanols, and Lipid Lowering

The intake of naturally occurring phytosterols and phytostanols is too small to lower serum and LDL cholesterol concentration even though these small doses can interfere with cholesterol metabolism [10]. This is the reason why phytosterols and phytostanols are added into foods and food supplements to obtain significant non-pharmacologic serum and LDL cholesterol reduction as part of heart healthy diet. Phytosterols and phytostanols 2 g/day significantly lower LDL cholesterol concentration by 8%–10% [9], irrespective of gender, age, ethnic background, body weight, background diet, or the cause of hypercholesterolemia, and even added to statin treatment. The 2 g phytosterol/phytostanol dose is the generally recommended daily dose for LDL cholesterol lowering by international guidelines at the moment (e.g., [11,12,13]).

Phytosterols and phytostanols interfere with intestinal cholesterol absorption. Several mechanisms for their inhibitory effect on cholesterol absorption have been suggested, e.g., by displacing cholesterol from mixed micelles (the micellar theory), by modifying the expression of genes encoding sterol transporter proteins Niemann-Pick C1-Like 1 (NPC1L1) or ATP binding cassette (ABCG5 and ABCG8) transporters promoting cholesterol efflux from enterocytes back into the intestinal lumen, by decreasing cholesterol re-esterification rate in the enterocyte, or by increasing cholesterol removal from the body via the transintestinal cholesterol efflux (TICE) pathway [14]. Of these different theories, only the micellar theory has gained experimental support [14,15,16,17,18]. It has been demonstrated that phytosterol or phytostanol intake was not able to activate the genes involved in intestinal sterol metabolism either in animal or in human studies [19,20], and the possible effect on TICE remains to be confirmed in human subjects.

If we look into the micellar theory in more detail, intestinal mixed micelles are globular aggregates of bile acids, fatty acids, monoglycerides and lysophospholipids. They act as a carrier for dietary and biliary cholesterol (and phytosterols/phytostanols and other non-cholesterol sterols) through the intestinal diffusion barrier onto the surface of the enterocyte. The mixed micelles serve cholesterol and non-cholesterol sterols to the NPC1L1 transporter. It has been demonstrated *in vitro* and in animal studies that the amount and degree of esterification of dietary phytosterols and phytostanols reduce cholesterol solubility and content in duodenal mixed micelles [15,16,17]. Furthermore, in healthy human subjects intestinal phytostanol ester perfusion studies demonstrated that at high intestinal phytostanol concentration cholesterol lost its micellar solubility, which resulted in a decreased intestinal absorption of cholesterol [18]. Consequently, less cholesterol will be transported to the liver [9]. For example, in postmenopausal women with coronary artery disease and primary moderate hypercholesterolemia without hypolipidemic treatment (mean serum cholesterol 6.0 mmol/L and mean serum triglycerides 1.4 mmol/L), phytostanol intake 3 g/day in esterified form decreased LDL cholesterol on average by 15%, reduced the absorption efficiency of cholesterol by −45%, increased the fecal elimination of cholesterol as neutral sterols by +45%, and stimulated the synthesis of cholesterol by 39% compared with the control period [21]. Interestingly, phytostanol consumption not only interferes with the absorption of cholesterol but also that of phytosterols so that in addition to serum and LDL cholesterol lowering, the serum phytosterol levels are reduced by about 30% during phytostanol intake [22].

The intake of added phytosterols and phytostanols have in general no effect on HDL cholesterol concentration. In some studies, phytosterols and phytostanols have modestly reduced serum triglyceride levels especially in those subjects with slightly increased baseline concentrations [23,24]. Lipoprotein (a) concentration remains unchanged [9,25], and phytostanols do not affect serum proprotein convertase subtilisin/kexin type 9 concentration [26].

## 2. Lipoprotein Metabolism

The studies discussed in the following are focused on clinical, randomized, controlled interventions during added doses of phytosterols or phytostanols. They include studies of VLDL, IDL, and LDL apoprotein (apo) B-100 and HDL apo AI kinetics [27,28,29], LDL particle size determination [27,30], analysis of small dense LDL cholesterol concentration [25,27,28,31], analysis of the concentrations of lipoproteins of different sizes [32,33], and evaluation of LDL lipidomics [30]. Postprandial lipids have also been evaluated [34,35,36,37].

### 2.1. Lipoprotein Kinetic Studies

Of the three lipoprotein kinetic studies, two were dealing with total and dense LDL apo B-100 and HDL apo AI kinetics with unstable isotopes [27,28], and the third with VLDL, IDL, and LDL apo B-100 and HDL apo AI kinetics with stable isotopes [29]. Table 1 presents the overall characteristics of the studies, study populations, and the results of LDL apo B-100 kinetics. In the two studies with type 2 diabetes [27,28], the glycemic balance was moderate (mean glycated hemoglobin 7.6% and 7.0%) and the subjects did not have insulin treatment. The studies were performed on normal habitual diet (mean dietary cholesterol and fat intakes 233 mg/day and 79 g/day). The third study population consisted of overweight–obese men with metabolic syndrome [29], and they consumed high cholesterol diet (cholesterol intake about 470 mg/day, total fat intake 102 g/day). Phytosterols and phytostanols were in esterified form in all these studies.

In the two studies with type 2 diabetes [27,28], phytostanol intake lowered LDL cholesterol by 9% and 14%, and LDL apo B-100 by 7% and 11% because of significantly reduced LDL apo B-100 transport rate by about 20% (Table 1). In addition, phytostanol lowered cholesterol and apo B-100 concentrations and the transport rate of apo B-100 also in the dense (1.037–1.055 g/mL) LDL fraction (*p* < 0.05 for all) [27]. Regarding the other lipoproteins, total and esterified cholesterol in VLDL and esterified cholesterol in IDL were significantly reduced by 12%, 13%, and 11%, respectively. HDL cholesterol was significantly increased by 11%, but the HDL apo AI kinetics remained unchanged [27]. Simultaneously, cholesterol absorption efficiency diminished by about 60% in both studies.

**Table 1:** Low density lipoprotein (LDL) kinetic studies.

Reference	Design	*N*	Dose, g/day	Duration, week	BMI, kg/m^2^	S-Chol, mmol/L	S-TG, mmol/L	LDL-C, mmol/L	LDL apo B-100, mg/dL	LDL apo B-100 TR, mg/kg/day	LDL apo B-100 FCR, pools/day
Ref [27]	RC PSA	11, T2D	3	6							
Control					26.5 ± 0.7	6.0 ± 0.2	2.1 ± 0.2	3.8 ± 0.2	65.2 ± 2.5	10.1 ± 0.5	0.339 ± 0.019
Intervention					26.4 ± 0.7	5.6 ± 0.2 *	2.1 ± 0.2	3.3 ± 0.2 *	60.5 ± 2.8 *	8.5 ± 0.1 *	0.314 ± 0.010
Ref [28]	RC PSA	8, T2D	3	7							
Control					26.6 ± 1.1	6.6 ± 0.1	2.4 ± 0.2	4.2 ± 0.1	83.1 ± 0.9	11.2 ± 1.0	0.321 ± 0.020
Intervention					28.4 ± 1.2	5.9 ± 0.2 *	2.4 ± 0.3	3.6 ± 0.1 *	74.2 ± 2.4 *	9.0 ± 0.5 *	0.305 ± 0.013
Ref [29]	RC PSE	9, MBO	2	4							
Control					35.5 ± 1.5	5.4 ± 0.5	1.8 ± 0.2	3.4 ± 0.4	121 ± 12	8.1 ± 0.6	0.34 ± 0.04
Intervention					35.3 ± 1.7	5.3 ± 0.5	1.9 ± 0.2	3.2 ± 0.4	109 ± 12	7.1 ± 0.6	0.30 ± 0.03

Mean ±standard error (SE). RC = randomized crossover; PSA = phytostanol ester; PSE = phytosterol ester; T2D = type 2 diabetes mellitus; MBO = metabolic syndrome; BMI = body mass index; S-Chol = serum cholesterol; S-TG = serum triglycerides; LDL-C =low density lipoprotein cholesterol; apo =apoprotein; TR = transport rate; FCR = fractional catabolic rate. To convert S-Chol mmol/L to mg/dL, please multiply with 38.6; to convert S-TG mmol/L to mg/dL, please multiply with 88.2; * significantly different from control period.

In the third study, no changes were observed in any of the serum or VLDL, IDL, LDL or HDL lipids, lipoprotein apo B-100 or apo AI concentrations, or in their kinetics shown for LDL apo B-100 in Table 1 [29]. Serum campesterol and sitosterol concentrations significantly increased suggesting that the subjects were consuming the phytosterol product, but it does not reveal how compliant they were. During added phytosterol intake, serum phytosterols cannot be used as biomarkers of cholesterol absorption. Unfortunately, cholesterol absorption efficiency was not evaluated in this study so that it remains open why the phytosterol consumption did not reduce LDL cholesterol concentration. Metabolic syndrome or overweight/obesity per se is not a reason for non-responsiveness [9].

If we compare the above results with kinetic studies performed with the other cholesterol absorption inhibitor, ezetimibe, which binds to the NPC1L1 receptor, the results are different [38,39]. In these two ezetimibe studies including 8 men with moderate primary hypercholesterolemia [38] and 15 obese subjects with central obesity and insulin resistance [39], ezetimibe significantly decreased LDL cholesterol concentration by 22% and 18%, and LDL apo B-100 concentration by 23% and 12%, respectively. The fractional catabolic rate of LDL apo B-100 increased by 24% and 29% with no change in LDL apo B-100 transport rate. Moreover, ezetimibe increased VLDL and IDL apo B-100 fractional catabolic rate but also the transport rate of VLDL apo B-100 [38]. The increased VLDL apo B-100 transport rate was assumed to result from the increased compensatory hepatic cholesterol synthesis by ezetimibe, which appears also during phytosterol and phytostanol consumption.

Cholesterol absorption inhibition reduces the hepatic cholesterol pool, which should activate the transcription factor sterol response element binding protein 2 (SREBP-2). Next, SREBP-2 upregulates *de novo* cholesterol synthesis and the LDL receptors [40], so that the fractional catabolic rate of LDL apo B-100 is expected to be increased. This, in fact, was observed during ezetimibe treatment.

Why, then, did the added phytosterols and phytostanols not increase the fractional catabolic rate of LDL apo B-100 [27,28,29]? Regarding the third study with no lipid, lipoprotein or apoprotein changes it can be expected that also the kinetic outcomes are unchanged [29]. The two other studies [27,28] consisted of type 2 diabetics. It has been demonstrated that poor glycemic control (glycated haemoglobin >7%) perturbs the LDL apo B-100 catabolism in type 2 diabetes [41], and hypertriglyceridemia even without overweight interferes with lipoprotein metabolism [42]. However, the glycemic control was good to moderate in the two studies at hand [27,28], the subjects were not obese (mean body mass index (BMI) about 26 kg/m^2^), and serum triglycerides were ≤2.5 mmol/L, suggesting that their metabolic variables were not too perturbed to affect the lipoprotein metabolism. In fact, the kinetic results of these two studies were practically similar to those obtained in non-diabetic subjects in our laboratory [43]. Furthermore, in these two studies cholesterol absorption was markedly inhibited and the whole-body cholesterol synthesis and its serum biomarkers were significantly increased. Accordingly, these results suggest that also the LDL receptors should have been upregulated. Considering the VLDL and IDL cholesterol reduction [27], it could be assumed that the LDL receptors, in fact, were upregulated, but they preferentially removed the large cholesterol-rich particles decreasing their conversion to LDL and thus lowering the LDL apo B-100 transport rate. This assumption is supported by unpublished results from another study in subjects with mild to moderate hypercholesterolemia (*n* = 92; BLOOD FLOW) [44], in which phytostanol ester consumption for six months reduced the small (27–35 nm) VLDL particles (mean ± SE 27 ± 3 *vs.* 21 ± 2 nmol/L, *p* = 0.021), but not those of LDL of any size or HDL (Data not shown). However, we have to admit that the limitations of the available lipoprotein kinetic studies during phytosterol and phytostanol supplementation is the small number of studies with limited number of subjects, who were all men and either diabetics or overweight/obese subjects with metabolic syndrome. We lack kinetic studies performed in primary moderate hypercholesterolemia, the target population for phytosterol and phytostanol consumption, and it would be interesting to study the LDL apo B-100 kinetics also in familial hypercholesterolemia, because phytosterols and phytostanols lower effectively LDL cholesterol concentration also in these subjects [9].

### 2.2. Lipoprotein Particle Size and Particle Concentrations

LDL particle size has been analyzed in subjects with type 2 diabetes without insulin therapy (Table 1) [27] and in overweight subjects (*n* = 32, mean BMI 27.8 kg/m^2^, mean serum cholesterol 5.6 mmol/L, mean serum triglycerides 1.4 mmol/L) [30]. Phytostanol (3 g/day) [27] and phytosterol 1.57 g/day [30] significantly reduced LDL cholesterol concentrations, but had no effect on LDL particle size in either of the studies. As comparison, ezetimibe did not either affect LDL particle size [38].

In four studies, the concentration of small dense LDL cholesterol has been analyzed [25,27,28,31]. Small dense LDL was separated enzymatically [25,31] or with ultracentrifugation (1.037–1.055 g/mL) [27,28]. In two studies, the subjects had type 2 diabetes and consumed phytostanols 3 g/day (Table 1) [27,28]. In the third study, adult subjects with metabolic syndrome (*n* = 108) consumed phytosterols 4 g/day for two months together with Western-type diet [31]. In the fourth study, hypercholesterolemic children (*n* = 25) aged 4.5–15.9 years consumed Step II diet and phytosterols 2 g/day for 6 to 12 months [25]. In all studies, phytosterol and phytostanol consumption significantly lowered both total (−13%, −9%, −14%, and −13%) and small dense LDL cholesterol concentrations (−18%, −16%, −21%, and −8%), respectively [25,27,28,31].

Changes in concentrations of different sized lipoproteins have been evaluated with nuclear magnetic resonance in subjects with metabolic syndrome and moderate hypertriglyceridemia (serum triglyceride levels about 2.2 ± 1.0 mmol/L (SD), *n* = 18) and in normolipidemic subjects (*n* = 75) in two studies lasting for two months [32]. In subjects with the metabolic syndrome, the phytostanol dose was 2 g/day, and 3.8–4.1 g/day in the normolipidemic subjects. In addition, in a large, randomized, controlled intervention the effect of phytosterols 2.5 g/day alone or combined with different doses of eicosapentaenoic + docosahexaenoic acids on 13 lipoprotein subclasses was evaluated [33]. The lipoprotein subclasses were analyzed with nuclear magnetic resonance, and with a computational model the lipoprotein particle turnover was estimated. Phytosterol consumption alone decreased the cholesterol content in total LDL and in all four LDL subclasses analyzed and also in most medium-sized VLDL particles with no shift in the LDL particle distribution. With the computational profiling phytosterols increased catabolic processes of IDL/LDL particles in relation to their influx. Phytosterol consumption did not affect HDL cholesterol content or HDL subclasses, or triglyceride contents in any of the subclasses. Adding eicosapentaenoic + docosahexaenoic acids to phytosterol reduced cholesterol and triglycerides in the large VLDL and small HDL particles, and increased in the large HDL particles, respectively.

In the subjects with the metabolic syndrome and moderate hypertriglyceridemia, phytostanols significantly reduced non-HDL cholesterol concentration by 14% and serum triglycerides by 27% compared with the control group [32]. The large (>60 nm) and medium (35–60 nm) sized VLDL particle concentrations, which quantitatively presented the smallest group of apo B-100 containing particles, were significantly reduced. Small VLDL, IDL, large and small LDL particles, and HDL particles were not affected by phytostanol intake. In normolipidemic subjects, phytostanol consumption reduced LDL cholesterol concentration by 14% compared with the control group, but had no effect on serum triglyceride concentration. Again, also the large VLDL particles were slightly but significantly reduced by phytostanols, but the reduction was smaller when compared to that observed in the subjects with metabolic syndrome. In addition, IDL particle (23–27 nm) concentrations were markedly reduced by phytostanols in this group.

The authors suggest that in subjects with elevated baseline serum triglyceride concentration phytostanol consumption reduces the hepatic production of the largest VLDL particles, which results in serum triglyceride lowering [32]. However, taking into consideration the results in normolipidemic subjects in the same study and the kinetic results in type 2 diabetes [27,28] and those with ezetimibe [38], it seems possible that the results can be interpreted also in a different way. It can be assumed that phytosterols and phytostanols simply enhance the LDL receptor activity, but the largest apo B-100 containing particles are preferentially removed. To resolve this question would need kinetic studies in subjects with combined hyperlipidemia and primary mild to moderate hypertriglyceridemia.

### 2.3. Postprandial Lipoproteins

Reduced cholesterol absorption can be anticipated to affect postprandial lipid metabolism. We evaluated in early studies the postprandial lipoprotein metabolism in 11 normolipidemic men by assessing postprandial concentrations of retinyl palmitate and squalene, validated biomarkers of postprandial lipoproteins [34], for 24 h. Two-week consumption of phytostanols 3 g/day as ester had no effect on postprandial cholesterol or triglyceride peak times or concentrations in plasma, chylomicrons, or VLDL, and similar results were obtained in a recent study in healthy subjects consuming phytostanols 4 g/day [37]. However, phytostanol ester reduced postprandial peak times, concentrations, and incremental areas under the curves for retinyl palmitate and squalene [34]. Evaluating the postprandial behavior of a large acute phytostanol load (1 g) with the test meal, it had no effect on serum cholesterol, triglyceride, α-tocopherol, β-carotene, or retinyl palmitate during the follow-up of 24 h [35]. However, serum campesterol concentration was significantly lowered starting from 6 h postprandially and reflecting the reduced sterol absorption. During long-term use of a large dose of phytostanols, avenasterol was postprandially reduced in chylomicrons [36] suggesting that chronic absorption inhibition with phytostanols decreases phytosterols in triglyceride-rich lipoproteins.

Accordingly, these results suggest that absorption inhibition did reduce postprandial lipoproteins, but did not affect the postprandial cholesterol or triglyceride values. Unfortunately, postprandial apo B-48 concentrations have not been evaluated during phytosterol and phytostanol consumption so that our knowledge of postprandial lipoprotein metabolism remains fragmentary and more information is warranted. Tremblay *et al.* [38] evaluated triglyceride-rich lipoprotein apo B-48 kinetics during ezetimibe therapy without any effects on the kinetic variables. Obviously, more information on postprandial lipoprotein metabolism is required.

## 3. Conclusions

The efficacy of phytosterols and phytostanols added to foods and food supplements to obtain significant non-pharmacologic serum and LDL cholesterol reduction is well documented, as well as the reproducibility of the effect in different populations regardless of age, gender, ethnic background, body weight, the cause of hypercholesterolemia, or background diet. Moreover, their role in cholesterol absorption and synthesis and their neutrality regarding HDL cholesterol metabolism is well characterized. According to inhibition of cholesterol absorption, they are expected to increase the removal of the circulating apo B-100 containing lipoproteins, but, in the few kinetic studies, only the reduced transport rate for LDL apo B-100 has been documented. In subjects with slightly elevated serum triglyceride levels in combination with mild to moderate hypercholesterolemia, e.g., in subjects with metabolic syndrome, phytosterol and phytostanol intake also reduces serum triglyceride levels. The triglyceride-lowering mechanism/s remain unresolved in detail, and they might have an important link to the overall control and assembly of hepatic lipids. Similarly, our knowledge of the postprandial lipoprotein and lipid metabolism (including also that of the phytosterols and phytostanols themselves) is too fragmentary to be able to construct a continuum from the postprandium to the whole-body lipoprotein metabolism. Finally, regarding the most important outcome of phytosterol and phytostanol supplementation, the reduction of atherosclerosis and its clinical implications, especially coronary heart disease, it would be important to combine the knowledge of lipoprotein metabolism to the tissue uptake and handling of different sterols.
